# Malignant transformation and tumour recurrence in sacrococcygeal teratoma: a global, retrospective cohort study

**DOI:** 10.1097/JS9.0000000000002045

**Published:** 2024-09-06

**Authors:** Lieke J. van Heurn, Joep P.M. Derikx, Nigel Hall, Jennifer H. Aldrink, Maria M. Bailez, Lohfa B. Chirdan, Shigehisa Fumino, Afua Hesse, Tuktu Soyer, Shawn StPeter, Jos Twisk, Tianyou Yang, Ernst L.W. van Heurn, The SCT-study consortium

**Affiliations:** a Department of Paediatric Surgery, Emma Children’s Hospital, Amsterdam UMC, University of Amsterdam and Vrije Universiteit Amsterdam; b Department of Epidemiology and Data Science, Amsterdam Public Health Research Institute, Amsterdam UMC, Vrije Universiteit Amsterdam, Amsterdam, The Netherlands; c Department of Surgery, Division of Paediatric Surgery, Nationwide Children’s Hospital, The Ohio State University College of Medicine, Columbus, Ohio; d Department of Surgery, Children’s Mercy Kansas City, Kansas City, USA; e Department of Surgery, Paediatric Surgery Unit, Jos University Teaching Hospital, Jos, Nigeria; f Department of Paediatric Surgery, Kyoto Prefectural University of Medicine, Kyoto, Japan; g Paediatric Surgery and Anatomy, Accra College of Medicine, Accra, Ghana; h Department of Paediatric Surgery, Faculty of Medicine, Hacettepe University, Ankara, Turkey; i Head of Surgical Department, Director of Surgical Simulation, Garrahan Childrens Hospital, Buenos Aires, Argentina; j University Surgery Unit, Faculty of Medicine, University of Southampton, Southampton, UK; k Department of Paediatric Surgical Oncology, Guangzhou Women and Children’s Medical Center, Guangzhou Medical University, Guangdong, People’s Republic of China

**Keywords:** malignant transformation, recurrence, sacrococcygeal teratoma

## Abstract

**Introduction:**

Sacrococcygeal teratoma (SCT) is a rare congenital tumour. The risk of malignancy and recurrence is not well defined. Previous studies are small and report differing conclusions about the timing of surgery and the duration of follow-up. The authors studied the risk of malignant transformation and SCT recurrence after surgery to address these gaps.

**Methods:**

This was a global retrospective cohort study. Data of consecutive SCT patients was obtained from 145 institutes in 62 countries. Malignant transformation, defined as malignancy at initial resection, malignant recurrence or death due to malignancy, and its risk factors were analysed.

**Results:**

Of the 3612 included patients, 3407 entered analysis. The risk of malignant transformation of the initial tumour was 3.3, 5.1, 10.1, and 32.9% at age 3 months, 6 months, 1 year, and 2 years, respectively. After 6 years, the censored risk of malignancy (64%) did not further increase. Recurrent SCT was diagnosed in 349 (10.2%) children with 126 (36.1%) malignant recurrences. Risk factors for recurrence were Altman type II [odds ratio (OR): 1.6, 95% confidence interval (CI): 1.2–2.2], Altman type III (OR: 1.6, 95% CI: 1.2–2.3), initial immature histology (OR: 1.9, 95% CI: 1.4–2.6), and initial malignant histology (OR: 4.0, 95% CI: 2.9–5.4).

**Conclusion:**

The risk of malignancy at initial resection in SCT increases with age reaching a plateau at 6 years of age. Recurrence after resection occurred in 10% of patients and 36% of these were malignant at that time. Altman type II or type III, and immature or malignant histology were associated with recurrence.

**Level of evidence:**

Level III.

## Introduction

Highlights

- This global retrospective cohort study includes 3612 sacrococcygeal teratoma patients from 62 countries worldwide.
- The risk of malignant transformation increases with age.
- The risk of recurrent sacrococcygeal teratoma after initial resection is 10%.
- If possible, complete resection of sacrococcygeal teratoma is recommended before age 1 month.
- Postoperatively, 6 years of follow-up is advised to detect possible recurrence.

Sacrococcygeal teratoma (SCT) is the most common neonatal tumour with a reported incidence of one per 14 000–35 000 live births^1^. The preferred treatment for SCT is complete resection at a young age. However, there is no generally accepted age at which resection should be done. Some clinicians advocate early surgery to minimise the risk of malignant tumour transformation as malignancy rates up to 70% have been reported if SCT is resected at age one year or older^2^. Others claim that surgery at a young age may lead to more operative and postoperative complications^3^.

Tumour recurrence after surgery occurs in two to 33% of patients and decreases the overall 10-year survival ranging from 60 to 92%^2,4,5^. Recurrent SCT is much more often malignant than SCT at initial resection with yolk sac tumour (YST) found in 22–56% of recurrences^1,6–8^. Definitive risk factors for tumour recurrence are relatively unknown, although tumour histology and completeness of resection, including coccygectomy, are reported as potential risk factors for recurrence^2,5^. However, most studied series are relatively small due to the rarity of the disease and the results are often contradictory. Furthermore, the duration of follow-up varies in the existing literature, which likely impacts the reported recurrence rate; although most recurrences present within 3 years after initial SCT resection, late recurrences up to 15 years have been described^1,5^. From a clinical perspective, there is no consensus regarding the appropriate duration of oncological follow-up of SCT patients.

The vast majority of the reported series of patients with SCT are from high-income countries (HICs) in which SCT patients are operated on within a few weeks after birth. The number of publications about SCT from low-income countries (LICs) and lower-middle-income countries (LMICs) is very limited^9,10^. Recently, unacceptable differences in mortality for congenital gastrointestinal anomalies have been shown in LICs, compared to upper middle-income countries (UMICs), and high-income countries (HIC’s)^11^. It is unknown if these differences are also present for SCT.

Given these uncertainties, we aimed to estimate the risk of malignant transformation of untreated SCT at different ages and, hence, the optimal time for resection. Furthermore, we aimed to identify factors associated with recurrent SCT and finally compare the treatment and outcome of SCT patients from different income countries.

## Methods

### Study design and participants

We did a global retrospective cohort study of SCT patients: ‘The SCT-study’ STROBE (The Strengthening the Reporting of Observational Studies in Epidemiology) and strengthening the reporting of cohort, cross-sectional, and case–control studies in surgery (STROCSS) (Supplemental Digital Content 1, http://links.lww.com/JS9/D406) 2021 guidelines were followed^12,13^.

We recruited as many patients from participating hospitals as possible regardless of geography. Paediatric surgeons and paediatric oncologists were invited to participate in the study through personal communication, the European Paediatric Surgeons’ Association (EUPSA) Network Office, PubMed publications, and a network of national and international study leads. Study information was provided in English, Spanish, French, and Russian. Participation was voluntary; no payment was made for data collection. Centres, which could include ten or more patients were invited to participate. An exception was made for centres from LICs and LMICs. For these centres, the minimum number of inclusions was set at five. The Supplementary Appendix (Supplemental Digital Content 2, http://links.lww.com/JS9/D407) provides an overview of the participating countries.

Due to the rarity of the condition, no sample size calculation was done and all eligible patients were included. Consecutive patients treated for SCT between 1982 and 2020 or a shorter time period in this era could be included into the study. Exclusion criteria were (a) born before 1982, (b) born after 2020, or (c) SCT as part of Currarino Syndrome (CS) as the risk of malignant transformation in (CS) may be reduced compared to ‘ordinary’ SCT^14,15^. The exact period of time for inclusions was determined by each individual participating centre as long as all consecutive patients in the chosen period were included.

All participating centres obtained local approval to participate in the study following their own legal and ethical regulations. Data transfer agreements were used to guarantee safe data use and storage.

Charts were reviewed by the individual local investigator. Data were validated with warning messages about possible errors when entered in Castor Electronic Data Capture (EDC). Furthermore, Castor files were structured so that out-of-range values could not be entered. Dependency fields were used for data and included initial resection, recurrence, death and follow-up. Furthermore, overall data distribution and frequencies in SPSS were checked to detect invalid entered data.

### Procedures

Data was anonymized by the local investigator to transform individual patient data into general anonymous information. Transformed patient data were uploaded with a personal link in Castor EDC and encrypted^16^. Every participating centre had only access to its own transformed patient data.

Included data was carefully selected by a group of experienced paediatric surgeons with interest in SCT treatment and was chosen based on main outcome variables used in previously published studies.

Collected data included generic and condition-specific variables. Generic variables included: country, sex (male/female/unknown), age at diagnosis (days), preoperative imaging modalities (none/ultrasound/computed tomography/MRI/unknown), initial tumour resection at the participating centre (yes/no/unknown), age at initial resection (days), outcome (survival/deceased/unknown), age at follow-up (days), age at death (days), and cause of death. Condition-specific variables were Altman classification (I/II/III/IV/unknown)^17^, CS (yes/no/unknown), initial SCT treatment (chemotherapy/surgery/no treatment/unknown), pathology (mature/immature/malignant/unknown), recurrence (yes/no/unknown), period between birth and recurrence (days), detection of recurrence (clinical examination/imaging/AFP/unknown), serum AFP-level at recurrence (μg/l), recurrent SCT pathology (mature/immature/malignant/unknown), and treatment of recurrent SCT (chemotherapy/surgery/no treatment/unknown).

Cause of death was collected as a free-text category. Participating country was used to categorise the country’s income status into LICs, LMICs, UMIC, and HICs according to the World Bank criteria^18^.

### Statistical analysis

Data are presented as mean with standard deviation (SD) if normally distributed and median with interquartile range (IQRs) if skewed; count data are presented as numbers and percentages. To assess malignant transformation of initial SCT and risk of recurrence over time, Kaplan-Meier curves were used.

Identification of factors associated with recurrent SCT was done by univariable and multivariable logistic regression analysis. All variables included in multivariable logistic regression analysis had maximum of 6.8% missing data. Sensitivity analysis was performed to analyse the effect of data imputation on the results of the multivariable logistic regression analysis.

Forward Wald selection was used to select variables significantly related to recurrent SCT. We report variables with the odds ratio (OR) and accompanying 95% confidence interval (CI).

Differences in patient demographics between country income were analysed with Fisher exact test for categorical variables, one-way ANOVA for normally distributed continuous variables, and Kruskal–Wallis test for non-normal continuous variables.

Statistical analyses were performed using SPSS for Windows version 25.0 software (SPSS) and Graph Pad Prism 8 (Graph Pad Software, Inc.). *P*<0.05 was considered statistically significant.

### Definitions

SCTs were classified according to the criteria proposed by the Surgical Section of the American Academy of Paediatrics^17^. Recurrence was defined as relapse of SCT at least 3 months after initial resection^15^. Recurrent SCT before 3 months is unlikely and is probably due to incomplete resection, therefore, these recurrences were excluded from data analysis. Malignancy-free survival was defined as time from birth to malignancy or death due to malignancy. Patients were censored at the number of days from birth to resection. In case of malignant recurrence or death due to malignancy, the number of days from birth to recurrence detection or death was used.

Variables investigated for recurrent SCT, with their respective categories, were Altman classification (I, II, III, and IV), histology (mature, immature, and malignant), age at diagnosis (days), income of the country (LIC, LMIC, UMIC, and HIC), and age at initial resection (days).

## Results

### Patient characteristics

In total, 145 centres from 62 countries treated 3612 patients for SCT. In 205 patients, SCT was associated with CS. These patients were excluded from the analysis. In total, 3407 patients were included in the study. Patient characteristics for the total population are described in Table 1.

**Table 1:** Patient characteristics for the total population and stratified per income countries.

	Total (*n*=3407)	High-income countries (*n*=2296)	Upper-middle income countries (*n*=677)	Lower-middle income countries (*n*=377)	Low-income countries (*n*=57)	*P*
Sex						
Male	844 (24.8%)	571 (24.9%)	161 (23.8%)	103 (27.3%)	9 (15.8%)	0.067
Female	2500 (73.4%)	1722 (75.0%)	491 (72.5%)	239 (63.4%)	48 (84.2%)	
Missing	63 (1.8%)	3 (0.1%)	25 (3.7%)	35 (9.3%)		
Median age at diagnosis, days	0 (0–54)	0 (0–17.5)	1 (0–108.5)	8 (0–150)	30 (2.5–556.5)	<0.001
Altman classification						
I	1036 (30.4%)	669 (29.1%)	227 (33.5%)	125 (33.2%)	15 (26.3%)	<0.001
II	1118 (32.8%)	753 (32.8%)	201 (29.7%)	137 (36.3%)	27 (47.4%)	
III	609 (17.9%)	407 (17.7%)	130 (19.2%)	63 (16.7%)	9 (15.8%)	
IV	548 (16.1%)	432 (18.8%)	851 (12.6%)	25 (6.6%)	6 (10.5%)	
Missing	96 (2.8%)	35 (1.5%)	34 (5.0%)	27 (7.2%)	–	
Median age at resection, days	13 (4–134.3)	9 (3–99)	17 (6–187.3)	40 (14–271.5)	52 (14.0–570)	<0.001
Pathology						
Mature	2168 (63.7%)	1504 (65.5%)	442 (65.3%)	184 (48.8%)	38 (66.7%)	0.004
Immature	625 (18.3%)	469 (20.4%)	91 (13.4%)	54 (14.3%)	11 (19.3%)	
Malignant	366 (10.7%)	245 (10.7%)	91 (13.4%)	27 (7.2%)	3 (5.3%)	
Missing	248 (7.3%)	78 (3.4%)	53 (7.8%)	112 (29.7%)	5 (8.8%)	
Recurrence						
Yes	349 (10.2%)	254 (11.1%)	56 (8.3%)	33 (8.8%)	6 (12.3%)	0.604
No	2829 (83.0%)	1967 (85.7%)	536 (79.2%)	276 (73.2%)	50 (87.7%)	
Missing	229 (6.8%)	75 (3.3%)	85 (12.6%)	68 (18.0%)	1 (1.8%)	
Median time between primary resection and recurrence, days	348 (196–666)	348 (200.5–679)	358 (182.5–673.8)	357 (195.5–668)	311.5 (131.5–654.5)	0.948
Recurrence pathology						
Mature	117 (33.5%)	96 (37.8%)	14 (25.0%)	7 (21.2%)	–	0.001
Immature	34 (9.7%)	23 (9.1%)	4 (7.1%)	7 (21.2%)	–	
Malignant	126 (36.1%)	91 (35.8%)	22 (39.3%)	7 (21.2%)	6 (100%)	
Missing	72 (20.6%)	44 (17.3%)	16 (28.6%)	12 (36.4%)	–	
Outcome						
Alive	2876 (84.4%)	2073 (90.3%)	534 (78.9%)	219 (58.1%)	50 (87.7%)	0.059
Death	140 (4.1%)	93 (4.1%)	25 (3.7%)	20 (5.3%)	2 (3.5%)	
Missing	391 (11.5%)	130 (5.7%)	118 (17.4%)	138 (36.6%)	5 (8.8%)	

Data are *n* (%) or median (IQR).

* *P* values represent univariable testing between country income strata.

Preoperative imaging was done with ultrasound in 595 (17.5%), CT in 280 (8.2%), and MRI in 526 (15.4%) patients, respectively. A combination of imaging modalities was performed in 1400 (41.1%) patients. In 606 children (17.8%), no imaging was performed or was unknown.

Surgery was the initial treatment in 2947 (86.5%) children. Surgery and chemotherapy were applied in 388 (11.4%) patients. Sixteen (0.4%) children received only chemotherapy. Furthermore, 13 (0.4%) children received no treatment and died immediately after birth due to bleeding or respiratory distress. In 43 (1.3%) children, treatment was unknown.

Overall for the entire cohort, the median follow-up was 5.1 years (IQR 2.3–9.2 years) after resection. In total, 140 (4.1%) patients died at a median age of 10.2 months (IQR 3 days–3.2 years). Ten years after the initial resection, 505 patients remained in follow-up. The 5-year survival was 95.4% and 10-year survival was 94.8%.

### Malignant transformation

**Figure 1:**
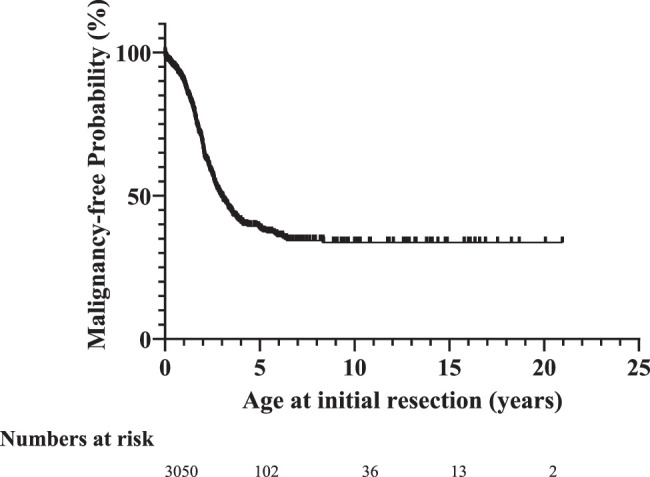
Malignancy-free probability at initial SCT resection.

Histological diagnosis after initial resection was mature teratoma in 2168 patients (63.7%), immature teratoma in 625 (18.3%), and malignant teratoma in 366 (10.7%). In 248 patients (7.3%), the histological diagnosis was unknown. The proportion of patients with malignant SCT increased with age. The probability of malignant transformation diagnosed at initial resection starts to increase directly after birth and increases further with age. The risk was 3.3, 5.1, 10.3, and 32.9% at 3 months, 6 months, 1 year, and 2 years, respectively (Fig. 1). After 6 years of age, the probability of initial malignant SCT did not further increase (64.2%). After 6 years, only one patient presented with an initial malignant SCT at the age of 8 years.

**Figure 2:**
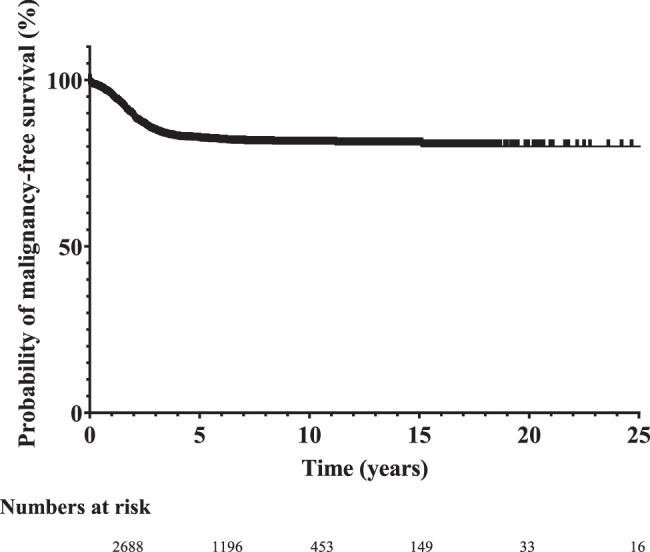
Probability of malignancy-free survival.

Malignancy-free survival including initial malignancies, malignant recurrences and deaths due to malignant disease, was 94.7% at age 1 year and 88.2% at 2 years (Fig. 2). Probability of overall malignancy-free survival remained relatively stable after the age of 6 years at 80.2%. After this period, four late malignancies were found; one initial malignancy, one malignant recurrence and two deaths due to malignancy. Late malignancies up to 15 years of age were found with an overall malignancy-free survival of 79.1%.

### Recurrent sacrococcygeal teratoma

**Figure 3:**
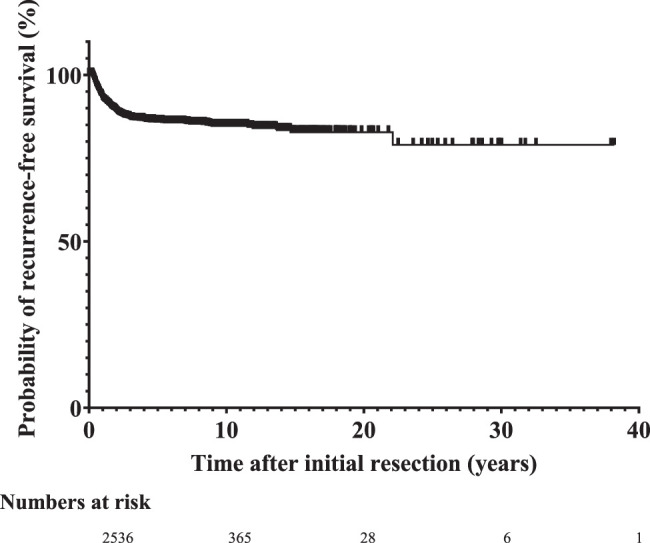
Probability of recurrence-free survival.

Three hundred forty-nine children (10.2%) developed recurrences at a median period of 11.4 months (IQR 6.4 months–1.8 years) after surgery. Ninety-six per cent of recurrences presented within the first 5 years after initial resection with a probability of recurrence-free survival after 5 years of 85.9% (Fig. 3). Late recurrences occurred up to 22.1 years after initial resection. Forty children died after recurrence at a median age of 15.6 months (IQR 6.2 months–2.3 years) after recurrence detection. Thirty died due to tumour progression or complications of chemotherapy. In 10 patients the cause of death was unknown. The other 284 survived; in 25 patients the outcome after recurrence was unknown.

Recurrent SCT was resected in 131 children, in 157 resections was combined with chemotherapy, and 33 children were treated with chemotherapy only. Four children received no treatment and treatment was unknown in 24 children.

The histology of recurrent SCT was mature teratoma in 117, immature teratoma in 34, and malignant teratoma in 126 children. The histology of recurrent SCT was unknown in 72 children. In 65 tumours (18.6%), there was a shift towards immaturity or malignancy.

### Income country

In this global study, 62 countries participated. Table 1 shows the main patient characteristics per income group. SCT diagnosis and resection were later in LICs compared to LMICs, UMICs and HICs. The median age at diagnosis was 30 days (IQR 2.5–556.5) in LICs, 8 (0–150) in LMICs, 1 (0–108.5) UMICs, and 0 (0–17.5) in HICs (*P*<0.001). The median age at resection was older: 52 days (IQR 14.0–570) in LICs compared to 40 (14–271.5), 17 (6–187.3), and 9 (3–99) days in LMICs, UMICs, and HICs, respectively (*P*<0.001). Despite the earlier diagnosis and resection, a higher proportion of malignant SCTs at initial treatment was found in HICs (*n*=245, 10.7%) and UMICs (*n*=91, 13.4%) compared to LMICs (*n*=27, 7.2%) and LICs (*n*=3, 5.3%) (*P*=0.004). The recurrence rates in all income groups were equivalent (*P*=0.604).

Recurrence histology differed between groups with a malignancy percentage of 100% in LIC compared to 21.2, 39.3, and 35.8% in LMICs, UMICs, and HICs, respectively (*P*=0.001). However, only six (12.2%) patients in LICs presented with recurrent SCT.

### Factors associated with recurrent SCT

Logistic regression analysis with forward selection using the Wald statistics was used to determine factors associated with recurrent SCT. Sensitivity analysis with multiple imputation showed no effect of the imputed data on the multivariable outcomes. Therefore, missing data imputation was not done. The final models showed a good fit according to the Hosmer and Lemeshow test with a value of 0.993 and a Concordance Index of 0.655 with the following variables being associated with recurrent SCT: Altman type II (OR 1.62, 95% CI: 1.18–2.23), Altman type III (OR 1.63, 95% CI: 1.23–2.35), immature pathology of the initial tumour (OR 1.91, 95% CI: 1.43–2.56), and malignant pathology of the initial tumour (OR 4.0, 95% CI: 2.91–5.41). Income of countries (*P*=0.760), age at diagnosis (*P*=0.254), and age at resection (*P*=0.073) were not associated with recurrence (Table 2).

**Table 2:** Risk factors for recurrent SCT.

		Univariable		Multivariable	
	Recurrence rate	Odds ratio	*P*	Odds ratio	*P*
Altman classification					
I	73 of 1036	1			
II	122 of 1118	1.631 (1.203–2.211)	0.002*	1.624 (1.180–2.234)	0.003*
III	76 of 609	1.900 (1.353–2.668)	<0.001*	1.627 (1.227–2.348)	0.009
IV	63 of 548	1.696 (1.189–2.419)	0.004	1.423 (0.967–2.093)	0.073
Income country					
Low	6 of 57	1			
Lower-middle	33 of 377	0.996 (0.397–2.502)	0.994		
Higher-middle	56 of 677	0.871 (0.357–2.121)	0.760		
High	254 of 2296	1.076 (0.457–2.535)	0.867		
Primary histology					
Mature	155 of 2168	1			
Immature	80 of 625	1.863 (1.3980–2.481)	<0.001*	1.911 (1.427–2.558)	<0.001*
Malignant	91 of 366	4.337 (3.245–5.795)	<0.001*	3.965 (2.906–5.411)	<0.001*
Median age at diagnosis (days)					
Yes	1 (0–248.3)				
No	0 (0-40)		0.254		
Median age at resection (days)					
Yes	17 (4–318)		0.073		
No	13 (4–120)				

## Discussion

This global, retrospective study provides information about the treatment and outcome of 3407 patients with SCT in 62 countries. We found that the risk of initial malignant transformation increases with age, with a malignancy rate of ~30% after 2 years. After 6 years, the risk of initial malignant transformation stabilized at 64%. Furthermore, due to the increasing risk of malignant transformation, initial resection is preferably done at a young age. However, young neonates and infants are at higher risk of operative and postoperative complications^3^. In the current study, information about gestational age at birth or operative and postoperative complications has not been collected, which could influence the optimal time for resection.

Overall malignancy-free survival was 94.7% at age 1 year and 88.2% at 2 years. This was higher than reported by others with 80 and 58% of malignancy-free survival at age 1 and 2 years, respectively^15^. However, the latter study defined malignancy-free survival as time from birth to malignancy or death. In the present study, only deaths due to malignant disease were included in the survival analysis. Malignancy-free survival stabilized after the age of 6 years at 80%.

In this study, 349 (10.2%) children developed recurrence after a median period of 11.4 months after initial resection with late recurrences up to 22 years after initial resection. Others have also described occasional late recurrences up to 15 years after surgery^1^. There is no consensus about the duration of follow-up after SCT resection and recommendation varies from 3 to 6 years after resection^5,19^. In the current study, 96% of the recurrences were found within 5 years after initial resection including all malignant recurrences.

Factors associated with SCT recurrence were Altman type II or type III and initial immature or malignant histology, which has been previously documented^5^. Other risk factors for recurrence described by others included incomplete resection, no coccyx removal, and tumour spillage^5,20^. In the current study, data of these risk factors was not collected.

### Income countries

Age at diagnosis and initial SCT resection were older in patients from LICs and LMICs compared to UMICs and HICs. This was also found in a recent study in children with retinoblastoma. This is probably due to late recognition and limited access to care^21^. In LIC Uganda only, it is estimated that only 15.2% of the Ugandan children with SCT presents in the hospital^22^. Moreover, the lack of paediatric intensive care facilities may force surgeons to postpone the SCT operation to an age with more physiological reserve.

In our study, the overall patient survival of children for all income countries was equivalent, which shows that SCT surgery can be done well and safely in LICs and LMICs. This is in contrast with others who found far better surgical outcomes for infants with other congenital disorders in HICs than in LICs or LMICs and found the income of the country as the greatest risk of mortality^11^.

The difference with our study results may be explained by the very small and selected proportion of SCT patients who present for surgery in LICs and LMICs^22^. In many LICs and LMICs, patients with large tumours might have died without reaching the hospital due to cultural believes or inability to access paediatric surgical care. Evidence for this presumption comes from the fact that many centres invited to participate in the study declined because they never resected a SCT. Furthermore, the proportion of LICs and LMICs with a traditionally good healthcare system such as Syria, was relatively high in our study.

Many SCT patients who present in hospitals in LICs and LMICs do not receive components of neonatal surgical care that are considered essential in HICs. It is estimated that only half of the hospitals, with paediatric surgery in West African countries have neonatal ICU and many countries have fewer than one paediatric surgeon per million children^9,11,23–25^.

### Limitations

This study has several limitations such as its retrospective study design and long inclusion period. The incidence of SCT is very low, therefore, it would take a very long time before a similar prospective dataset could be obtained. Secondly, the number of collected variables was limited. This may have facilitated participation. Most variables could be obtained from electronic data files or patient letters. The percentage of missing data used in the analyses was, therefore, small, with a maximum percentage of 6.8%. Thirdly, the use of anonymized data made data validation not possible. On the other hand, this also may have reduced the risk of selection bias by not sharing data with an unfavourable outcome. Finally, there is bias because an unknown proportion of patients has not been included in the study. In an unknown but probably a relatively small number of patients in Western countries, the pregnancy of SCT patients is discontinued. Many SCT patients in LICs and LMICs do not receive any treatment.

### Recommendations

We recommend complete resection of SCT before 1 month of age to minimise the risk of malignant transformation as long as it is safe and feasible. Furthermore, we advise to follow-up to 6 years of age since the vast majority of recurrences present within this timeframe. After this period, the chance of malignant transformation is probably small.

## Conclusion

The United Nations encourages nations to create networks of experts for rare diseases and to increase support for research, by strengthening international collaboration and coordination of research efforts and the sharing of data, while respecting its protection and privacy^26^. We have shown that it is feasible to set up a large global study for a rare disease. The comprehensive study design herein and the large patient cohort enabled the identification of risk factors for recurrent SCT and essential information regarding malignant SCT transformation. With large, shared patient data sets, it is possible to answer important clinical questions that cannot be answered otherwise in order to improve the quality of care in every part of the world.

## The SCT-study consortium

Siffredi Juan Ignacio (Department of Pediatric Surgery, Hospital Garrahan Buenos Aires, Argentina); Pablo Lobos (Hospital Italiano de Buenos Aires, Argentina); Holger Till (Head of the Dept. of Paediatric and Adolescent Surgery, Medical University of Graz, Austria); Ashrarur Rahman Mitul (Bangladesh Shishu Hospital & Institute, Dhaka, Bangladesh); Olga Govorukhina (Center of Pediatric Surgery of Belarus); Natalya Prokopenya (Center of Pediatric Surgery of Belarus); Antoine De Backer (Universitair Ziekenhuis Brussel and Saffier Network for Rare Diseases in Pediatric Surgery, Belgium); Helena Reusens (Department of Pediatric Surgery, Hôpital des Enfants Reine Fabiola, Université Libre de Bruxelles); Simone de Campos Vieira Abib (Pediatric Oncology Institutute - GRAACC - Federal University of São Paulo, Brazil); Penka Peneva Stefanova-Peeva (Department of Pediatric surgery, University Hospital "St. George" and Medical University, Plovdiv, Bulgaria); Nadezhda Tolekova (Pediatric Surgery department of UMHATEM "N. I. Pirogov" Sofia, Bulgaria); Mouafo Tambo Faustin (Yaounde Gynaeco-Obstetric and Pediatric Hospital-faculty of medecine and biomedical sciences- University of Yaounde Cameroon); Jean-Martin Laberge (McGill University, Montreal Children’s Hospital – Shriners Hospital for Children Canada); Augusto Zani (Division of General and Thoracic Surgery, The Hospital for Sick Children, Toronto, ON, Canada); Richard J.B. Walker (Division of General and Thoracic Surgery, The Hospital for Sick Children, Toronto, Canada; Division of General Surgery, Department of Surgery, University of Toronto, Toronto, Canada); Maricarmen Olivos Pérez (Hospital de Niños Dr. Roberto del Rio. Santiago, Chile); Marco Andrés Valenzuela (Hospital de Niños Dr. Roberto del Río. Santiago, Chile); Yuanchao Shen (Department of Pediatric Surgery, Guangzhou Women and Children’s Medical Center, Guangzhou Medical University, China); Yan Zou (Department of Pediatric Surgery, Guangzhou Women and Children's Medical Center, Guangzhou Medical University, Guangzhou,510623, China); Stanko Ćavar (Division pediatric surgery, University Hospital centre Zagreb, Croatia); Zenon Pogorelić (Department of Pediatric Surgery, University Hospital of Split and Department of Surgery, University of Split, School of Medicine, Croatia); Lucie Pos (Department of Paediatric Surgery, 2nd Faculty of Medicine, Charles University and University Hospital Motol, V Uvalu 84, Prague 5, 150 06, Prague, Czech Republic); Richard Skaba (Department of Paediatric surgery, 2nd Faculty of Medicine, Prague, Czech Republic); Peter Hjorth Jørgensen (Department of Pediatric Surgery, Rigshospitalet Copenhagen University Hospital, Copenhagen, Denmark); Amr Abdelhamid AbouZeid (Faculty of medicine, Department of Pediatric Surgery; Ain Shams University, Cairo, Egypt); Mahmoud Elfiky (Kasr Al Ainy Faculty of Medicine, Cairo University); Heba Taher (Pediatric Surgery Cairo University, Egypt); Matis Märtson (Tallinn Children's Hospital, Head of the Surgical Clinic, Tallinn, Estonia); Miliard Derbew (Medical Education Partnership Initiative Junior Faculty Project, School of Medicine, College of Health Sciences, Addis Ababa University, Ethiopia); Workye Molla Tigabie (St.Peter Specialized Hospital, Addis Ababa, Ethiopia); Antti Koivusalo (New Children´s Hospital, (Section of Pediatric Surgery, University of Helsinki), Helsinki, Finland); Mikko Pakarinen (Section of Pediatric Surgery, Pediatric Liver and Gut Research Group, New Children’s Hospital, University of Helsinki and Helsinki University Hospital, Helsinki, Finland, and Department of Women’s and Children’s Health, Karolinska Institute, Stockholm, Sweden); Olivier Abbo (Surgical Department, Hôpital des Enfants de Toulouse, France); Alexis Pierre Emmanuel Arnaud (Department of pediatric surgery, CHU Rennes, Univ Rennes, Rennes, France); Quentin Ballouhey (Department of Pediatric Surgery, Limoges University Hospital Center, Limoges, France); Francois Bastard (University Hospital, Angers, France); Anne Dariel (Pediatric surgery department, Hospital for sick children La Timone, Assistance publique des hôpitaux de Marseille, Marseille, France); Sabine Irtan (Sorbonne Université, Armand Trousseau Hopsital –Assistance Publique Hôpitaux de Paris, Paris, France); Jean Francois Lecompte (Pediatric Surgery Department, Hopitaux Pédiatriques de Nice CHU-Lenval, Nice, France); Guillaume Levard (Pediatric Surgery Department, University Hospital, Poitiers); Sabine Sarnacki (Department of Pediatric Surgery, Hôpital Necker-Enfants Malades - APHP GH Centre and Université de Paris Cité, Paris, France); Rony Sfeir (Pediatric Surgery Unit, University Hospital of Lille Jeanne de Flandre, Lille, France); Nicolas Vinit (Department of Pediatric Surgery and Urology, Necker-Enfants Malades Hospital, APHPand Université Paris Cité, Paris, France); Anne-Sophie Holler (Department of Pediatric Surgery, Dr. von Hauner Children's Hospital, University Hospital, LMU Munich); Marietta Jank (Department of Pediatric Surgery, University Medical Center Mannheim, Heidelberg University, Mannheim, Germany); Martin Lacher (Head of Department of Pediatric Surgery, University of Leipzig, Leipizig, Germany; Oliver J. Muensterer (Department of Pediatric Surgery, Dr. von Hauner Children's Hospital, University Hospital, LMU Munich, Germany); Karin Rothe (Department of Pediatric Surgery, Charité Universitätsmedizin Berlin, corporate member of Freie Universität Berlin and Humboldt Universität zu Berlin, Germany); Udo Rolle (University Hospital Frankfurt/M., Department of Paediatric Surgery and Paediatric Urology); Zoi Lamprinou (P. & A. Kyriakou Children’s Hospital, Athens, Greece); Zsuzsanna Jakab (Hungarian Childhood Cancer Registry, 2nd Department of Pediatrics, Semmelweis University, Budapest, Hungary); Peter Vajda (Division of Paediatric Surgery, Medical School, University of Pécs, Hungary); Agnes Vojcek (Division of Pediatric Hematology and Oncology, Department of Pediatrics, University of Pécs Medical School, Pécs, Hungary); Kenneth KY Wong (Division of Paediatric Surgery, Department of Surgery, Li Ka Shing Faculty of Medicine, University of Hong Kong, Queen Mary Hospital, 102 Pokfulam Road, Hong Kong, Hong Kong); Mohan K. Abraham (Department of Pediatric surgery and Pediatric urology, Amrita Institute Of medical sciences, Kochi, Kerala, India); Kirtikumar J Rathod (Department of Pediatric Surgery, All India Institute of Medical Sciences, Jodhpur, India); Shilpa Sharma (Department of Pediatric Surgery, All India Institute of Medical Sciences, New Delhi); Gunadi (Pediatric Surgery Division, Department of Surgery, Faculty of Medicine, Public Health and Nursing, Universitas Gadjah Mada, Dr. Sardjito Hospital, Yogyakarta, Indonesia); Mehdi Sarafi (Pediatric Surgery Research Center, Research Institute for Children’s Health, Shahid Beheshti University of Medical Sciences, Teheran, Iran); Ahmad Khaleghnejad Tabari (Pediatric Surgery Research Center, Research Institute for Children's Health, Shahid Beheshti University of Medical Sciences, Tehran, Iran); Muataz Al Ani (Ninevah college of Medicine, Alkhansaa teaching hospital, Mosul pediatric surgery centre, Iraq); Gavin Kane (Department of Paediatric Surgery, Children's Health Ireland at Crumlin, Dublin, Ireland); Igor Sukhotnik (Department Pediatric Surgery, Tel Aviv Sourasky Medical Center, Tel Aviv University, Israel); Stefano Avanzini (Pediatric Surgery Department, IRCCS Istituto Giannina Gaslini, Genoa, Italy); Pietro Bagolan (Area of Fetal, Neonatal, and Cardiological Sciences, Children's Hospital Bambino Gesù-Research Institute, Rome, Italy; Department of Systems Medicine, University of Rome "Tor Vergata", Rome, Italy); Piergiorgio Gamba (Pediatric Surgery, Department of Women's and Children's Health, University of Padua, Padua, Italy); Riccardo Guanà (Pediatric and Neonatal Surgery Unit, Regina Margherita Children’s Hospital, Turin, Italy); Alessandro Inserra (Tor Vergata University of Rome, Director of the graduate school in Pediatric Surgery; Academic Director of Pediatric Surgery, Head of U.O.C.General and Thoracic Surgery, Bambino Gesu' Pediatric Hospital); Mario Lima (Pediatric Surgery Sant'Orsola Hospital, IRCSS, University of Bologna, Bologna, Italy); Antonino Morabito (Meyer Children's Hospital IRCCS Florence, University of Florence, Italy); Alessandro Raffaele (Department of Pediatric Surgery, Fondazione IRCCS Policlinico San Matteo, Pavia, Lombardia, Italy); Giovanna Riccipetitoni (Fondazione IRCCS Policlinico San Matteo, Pavia, Italy); Calogero Virgone (Pediatric Surgery, Department of Women's and Children's Health, University of Padua, Padua, Italy); Yapi Landry Ake (Cocody Teaching hospital at Abidjan in Ivory Coast ; Department of Mother and Child health, pediatric surgery); Yoshiaki Hirohata (Department of Pediatric Surgery, Kyoto Prefectural University of Medicine, Kyoto, Japan); Maho Inoue (Department of Surgery, Saitama Children's Medical Center, Saitama, Japan); Yutaka Kanamori (Department of General Surgery, National Center for Child Health and Development, Tokyo, Japan); Naonori Kawakubo (Department of Pediatric Surgery, Graduate School of Medical Sciences, Kyushu University, Fukuoka, Japan); Takashi Sasaki (Department of Pediatric Surgery, Osaka City General Hospital, Osaka, Japan); Tomoaki Taguchi (Department of Pediatric Surgery, Graduate School of Medical Sciences, Kyushu University, Fukuoka, Japan); Tatsuro Tajiri (Department of Pediatric Surgery, Kyoto Prefectural University of Medicine, Kyoto, Japan); Hirofumi Tomita (Department of Surgery, Tokyo Metropolitan Children's Medical Center, Tokyo, Japan); Noriaki Usui (Department of Pediatric Surgery, Osaka Women's and Children's Hospital, Izumi, Japan); Mohit Kakar (Department of Pediatric Surgery, Riga Stradins University & Children's Clinical University Hospital, Riga, Latvia); Vidmantas Barauskas (Vaikų chirurgijos klinika, Lietuvos sveikatos mokslų universiteto ligoninė Kauno klinikos, Eivenių g. 2 LT-50161, Kaunas); Toni Risteski (University Clinic for Pediatric Surgery, Faculty of Medicine, Ss. Cyril and Methodius, University in Skopje, N Macedonia); Dayang Anita Abdul Aziz (Paediatric Surgery Unit, Department of Surgery, Faculty of Medicine, Universiti Kebangsaan Malaysia, Kuala Lumpur, Malaysia); Mohd Yusran Othman (Hospital Tunku Azizah, Kuala Lumpur Women's and Children's Hospital, Malaysia); Jose Martin Palacios Acosta (Servicio de Cirugía Oncológica, Instituto Nacional de Pediatría, Mexico City, Mexico); Marija Kolinovic (Pediatric Surgeon, Institute for children's diseases, Clinical Centre of Montenegro, Podgorica, Montenegro); Mohammed Oulad Saiad (Department of general pediatric surgery, Mother and child unit, university hospital Mohamed VI, Cadi Ayyad University, Marrakesh, Morocco); Robertine van Baren (Department of Surgery and Pediatric Surgery, University Medical Center Groningen, Groningen, Netherlands); Ivo de Blaauw (Department of Pediatric Surgery, Radboud University Medical Center, Amalia Children's Hospital, Nijmegen, The Netherlands); Wim G. van Gemert (Department of Pediatric Surgery, University Medical Centre Maastricht, Maastricht, the Netherlands); Ramon Gorter (Emma Children’s Hospital, Amsterdam UMC, location University of Amsterdam, Paediatric Surgery, Amsterdam, The Netherlands); Marijke E.B. Kremer (Department of Pediatric Surgery, University Medical Centre Maastricht, Maastricht, the Netherlands); Cornelius E.J. Sloots (Department of Pediatric Surgery, Erasmus MC Sophia Children’s Hospital, Rotterdam, The Netherlands); Marc Wijnen (Department of Pediatric Surgery, Radboud University Medical Center, Amalia Children's Hospital, Nijmegen, The Netherlands & Prinses Maxima MC); Rene M.H. Wijnen (Department of Pediatric Surgery and Intensive Care, Erasmus MC-Sophia Children's Hospital, Rotterdam, Netherlands); Marieke J. Witvliet (Department of Pediatric Surgery, University of Utrecht, Wilhelmina Children's Hospital, UMC Utrecht, Utrecht, The Netherlands); Adesoji Ademuyiwa (Paediatric Surgery Unit, Department of Surgery, Faculty of Clinical Sciences, College of Medicine, University of Lagos. Idi Araba, Lagos, Nigeria; Paediatric Surgery Unit, Department of Surgery, Lagos University Teaching Hospital. Idi Araba, Lagos, Nigeria); Chris Bode (Paediatric Surgery Unit, Department of Surgery, Faculty of Clinical Sciences, College of Medicine, University of Lagos. Idi Araba, Lagos, Nigeria; Paediatric Surgery Unit, Department of Surgery, Lagos University Teaching Hospital. Idi Araba, Lagos, Nigeria); Okechukwu Hyginus Ekwunife (Nnamdi Azikiwe University Teaching Hospital Nnewi, Nigeria); Justina Seyi-Olajide (Paediatric Surgery Unit, Department of Surgery, Lagos University Teaching Hospital. Idi Araba, Lagos, Nigeria); Aminu Muhammed Umar (Abubakar Tafawa Balewa University Teaching Hospital, Bauchi, Nigeria); Kristin Bjornland (Department of pediatric surgery, Oslo University Hospital, postboks 4950 Oslo, Norway and University of Oslo); Muhammad Arshad (Professor of Pediatric surgery, Liaquat National Hospital and Aga khan university, Karachi Pakistan); Mohammad Ajad Chaudhry (Department of pediatric surgery, Children Hospital, Shaheed Zulfiqar Ali Bhutto Medical University, Islamabad, Pakistan); Muhammad Bilal Mirza (University of Child Health Sciences Lahore Pakistan); Beda Espineda (Philippine Children's Medical Center as a Senior Consultant in Department of Pediatric Surgery); Maria Celine A. Villegas (Chief, Division of Pediatric Surgery, Department of Surgery, University of the Philippines - Philippine General Hospital; Associate Professor, University of the Philippines College of Medicine, University of the Philippines Manila); Weronika Jaron (Department of Pathology, The Children's Memorial Health Institute, Warsaw, Poland); Piotr Kalicinski (Department of Pediatric Surgery and Organ Transplantation, Children's Memorial Health Institute, 04-730 Warsaw, Poland); Maciej Murawski (Department of Pediatric Surgery and Urology, Medical University of Gdansk, Poland); Rui Alves (Pediatric Surgery Department, Hospital Dona Estefania, Lisboa, Portugal), Maria Carolina Sobral (Hospital Dona Estefânia - Centro Hospitalar Universitário Lisboa Central); Vlad-Laurentu David (Department of Pediatric Surgery and Orthopedics, "Victor Babes" University of Medicine and Pharmacy Timisoara, Romania); Alexey A. Gusev (FSAI "NMRC of Сhildren Health" MHRF, Moscow, Russian Federation; RUDN University, Moscow, Russian Federation); Khvorostov I.N. (Volgograd State Medical University); Yury Kozlov (Irkutsk Regional Children’s Hospital, Director of Regional Center for Pediatric Minimally Invasive Surgery and Pediatric Robotics, Irkutsk, Russia; Department of Pediatric Surgery, Irkutsk State Medical Academy of Postgraduate Education, Russia; Department of Pediatric Surgery and Pediatrics, Irkutsk State Medical University, Russia); Minaev Sergey Viktorovich (Department of pediatric surgery of Stavropol State Medical University, Russia); Maja Milickovic (Department of Abdominal Surgery, Institute for Mother and Child Healthcare of Serbia "Dr Vukan Cupic", Belgrade, Serbia); Sanja Sindjic-Antunovic (University Children’s Hospital, Center for Pediatric Surgery and Medical Faculty University of Belgrade, Serbia); York Tien Lee (Senior Consultant, Department of Paediatric Surgery, KK Women’s and Children’s Hospital, Singapore; Clinical Assistant Professor, DUKE-NUS Medical School, Singapore); Rebeka Pechanová (National Institute of Children´s Diseases, Bratislava, Slovakia); Jože Maučec (Department for Pediatric Surgery, University Medical centre Ljubljana, Slovenia); Hyun-Young Kim (Department of Pediatric Surgery, Seoul National University Hospital, 101 Daehak-ro, Jongno-gu, Seoul, 03080, Republic of Korea; Department of Pediatric Surgery, Seoul National University College of Medicine, Seoul, Republic of Korea); Seong Chul Kim (Department of Pediatric Surgery, University of Ulsan College of Medicine and Asan Medical Center, Seoul, Korea); Sohyun Nam (Division of Pediatric surgery, Department of Surgery, Inje University Busan Paik hospital, Busan, South Korea); Gabriela Guillén (Department of Pediatric Surgery, University Hospital Vall d´Hebron, Barcelona, Spain); Leopoldo Martinez (General-Oncologic Pediatric Surgery, Children's Hospital La Paz, Spain); Maria Molina (Virgen del Rocio Children's Hospital, Department of Pediatric Surgery, Sevilla, Spain); Fernando Vázquez Rueda ( Pediatric Oncologic Surgery, Pediatric Surgery Service, Hospital Universitario Reina Sofía. Córdoba, Spain); Oscar Girón Vallejo (Pediatric Surgery Department. Pediatric Surgical Oncology, Virgen de la Arrixaca University Clinical Hospital, Murcia, Spain); Maria Bordallo Vazquez (Pediatric Surgery Department. Pediatric Surgical Oncology Unit. La Fe University and Polytechnic Hospital, Valencia, Spain); Naveen Wijekoon (Department of Surgery, University of Colombo, Consultant Paediatric Surgeon - Lady Ridgeway Hospital, Sri Lanka); Mette Hambraeus (Department of Pediatric surgery, Skane University Hospital Lund, Lund, Sweden); Helene Engstrand Lilja (Department of Women’s and Children’s Health | Karolinska Institutet, Unit of Pediatric Surgery | Karolinska University Hospital); Par-Johan Svensson (Consultant Paediatric Surgeon Department of Paediatric Surgery, Astrid Lindgren´s Childrens Hospital, Karolinska Hospital and Karolinska Institute, Stockholm, Sweden); Tomas Wester (Karolinska University Hospital, Stockholm, Sweden); Husam Dalati (Department of Pediatric Surgery, Children Hospital, Damascus, Syria); Houssain Al Halabi (Department of Pediatric Surgery, Children Hospital, Damascus, Syria); Qusai Mashlah (Department of Pediatric Surgery, Children Hospital, Damascus, Syria); Shih-Hsiang Chen (Division of Hematology/Oncology, Department of Pediatrics, Chang Gung Memorial Hospital, Chang Gung University College of Medicine, Taoyuan, Taiwan); Montinee Supchatura (Department of Surgery, Queen Sirikit National Institute of Child Health, Bangkok, Thailand); Saloua Ammar (Département of Pediatric surgery, Hedi Chaker Hospital University of medecine of Sfax, University of Sfax, Tunisia); Salma Mani (Département of Pediatric surgery, Fattouma Bourguiba Hospital, University of medecine of Monastir, Tunisia); Orkan Ergün (Ege University Department of Pediatric Surgery, Izmir, Türkiye); Cigdem Ulukaya Durakbasa (Department of Pediatric Surgery, Istanbul Medeniyet University Faculty of Medicine); Samir Hasan (Ege University Department of Pediatric Surgery, Izmir, Türkiye); Ayşe Karaman (University of Health Sciences Turkey, Dr Sami Ulus Maternity and Children Health and Research Application Center, Department of Pediatric Surgery); Ibrahim Karaman (University of Health Sciences Turkey, Dr Sami Ulus Maternity and Children Health and Research Application Center, Department of Pediatric Surgery); Tezer Kutluk (Department of Pediatric Oncology, Hacettepe University, Faculty of Medicine & Cancer Institute Ankara, Turkey); Yevhen Rudenko (Department of Pediatric Surgery, Bogomolets National Medical University, Neonatal Surgery unit, National Specialized Children Hospital “Ohmatdyt“, Kyiv, Ukraine); Sarah Braungart (Department of Paediatric Surgery, Leeds Teaching Hospitals, UK); Deepika Bhojwani (Department of paediatric surgery, Cambridge University Hospitals NHS Foundation Trust); Alison Morag Campbell (Department of paediatric surgery, Great North Children's Hospital, Newcastle); Manal Dhaiban(FRCS England, MS Paediatric surgery, Speciality Registrar Paediatric surgery in Birmingham Children hospital UK); Paul Farrelly (Department of Paediatric Surgery, Royal Manchester Children's Hospital, Manchester, UK); Florian Friedmacher (Department of Pediatric Surgery, The Royal London Hospital, London, United Kingdom; Department of Pediatric Surgery, University Hospital Frankfurt, Goethe University Frankfurt, Germany); Robin Garrett-Cox (Consultant Paediatric Surgeon at Bristol Royal Children's Hospital, Bristol UK); Stefano Guiliani (Developmental Biology and Cancer Programme, UCL Great Ormond Street Institute of Child Health, London, UK; Department of Specialist Neonatal and Paediatric Surgery, NHS Foundation Trust, Great Ormond Street Hospital for Children, London, UK); Paul Losty (Professor of Paediatric Surgery, Institute Of Life Course And Medical Sciences, University Of Liverpool, UK); Jonathan Neville (Department of Paediatric Surgery, University Surgery Unit, University Hospitals Southampton); Kathryn O'Shea (Royal Manchester Children's Hospital, Manchester, UK); Rebecca A Roberts (Department of Paediatric Surgery and Urology, Bristol Royal Hospital for Children, Bristol); Mohamed Shalaby (Department of Pediatric Surgery, Royal Hospital for Sick Children, Edinburgh, United Kingdom); G. Suren Arul (Consultant paediatric surgeon, Birmingham Children’s Hospital, Birmingham, UK); Zachary Kastenberg (Division of Pediatric Surgery, Department of Surgery, University of Utah School of Medicine, Salt Lake City, USA); Maria E. Knaus (Department of Surgery, University of Tennessee Health Science Center); Bethany J. Slater (Department of Surgery, University of Chicago, Chicago, IL); Bruce Bvulani (Department of Paediatric Surgery, University Teaching Hospital of Lusaka, Lusaka, Zambia)

## Ethical approval

The Medical Ethical Board of Amsterdam University Medical Centre (Amsterdam UMC), determined that Medical Research Involving Human Subject Act (WMO) does not apply to the study and that official approval of the committee was not required (reference number W19_329 # 19.388).

## Consent

Individual patient written consent was not obtained since the study included patients with intrauterine and neonatal death. Patient data collection was fully anonymous and cannot be traced back to the individual patient. The absence of written consent was discussed with the Medical Ethical Board.

## Source of funding

This study received funding from Kika Children Cancer-free Foundation. Kika Children Cancer-free Foundation had no involvement in collection, analysis and interpretation of data or in writing and decision to submit the manuscript for publication.

## Author contribution

L.W.E.v.H.: gained study funding, wrote the study protocol, and established the SCT-study consortium; L.J.v.H.: designed the data collection forms, coordinated the data collection and validation, undertook the data analysis, and wrote and revised the manuscript; the writing committee (J.H.A., M.M.B., L.B.C., J.P.M.D., S.F., N.J.H., A.H., L.J.v.H., L.W.E.v.H., T.S., S.StP., J.T., and T.Y.) contributed to the data interpretation, manuscript content, and revisions; L.J.v.H. and J.T.: statistical analysis; L.W.E.v.H., L.J.v.H., J.P.M.D., and statistician (J.T.) had full access to all the study data; L.J.v.H. and statistician (J.T.) validated the data. All authors (SCT-study group) approved the manuscript and had final responsibility for the decision to submit for publication. All authors were local investigators. Local investigators gained local study approval, used the protocol to identify eligible patients, collected data, entered data in Castor EDC, and checked the data to prevent duplicate entries and ensure accuracy. All authors have read and approved the final manuscript.

## Conflicts of interest disclosure

All authors declare no conflicts of interest

## Research registration unique identifying number (UIN)

ISRCTN Registry ISRCTN17493302 Malignant transformation and tumour recurrence in sacrococcygeal teratoma, a congenital disorder in newborns. Retrospectively registered on 28 April 2024.

## Guarantor

L.W.E. van Heurn, J.P.M. Derikx, and L.J. van Heurn are guarantors for this study.

## Data availability statement

Following publication of the study results, the full, anonymous de-identified patient dataset will be made available. Data requestors will need to sign a data access agreement.

## Provenance and peer review

Not commissioned, externally peer-reviewed.

## Supplementary Material
