# Supplementary material for: Malignant transformation and tumour recurrence in sacrococcygeal teratoma: a global, retrospective cohort study
Source: Int J Surg. 2024 Sep 6;110(11):7177–86. doi: 10.1097/JS9.0000000000002045 (PMC11573091; doi:10.1097/JS9.0000000000002045)
Supplement: Supplementary file 2 [file js9-110-7177-s002.docx]

**Supplementary appendix**

Supplement to:

Malignant transformation and tumour recurrence in sacrococcygeal teratoma: a global, retrospective cohort study

**Malignant transformation and tumour recurrence in sacrococcygeal teratoma: a global, retrospective cohort study**

**Appendix Supplement 1: Authorship (all co-authors PubMed citable)**

**Principal Investigator**

Lodewijk Willem Ernest van Heurn (Emma Children’s Hospital, Amsterdam UMC, location University of Amsterdam, Paediatric Surgery, Amsterdam, The Netherlands)

**Writing Committee**

Jennifer H. Aldrink (Division of Pediatric Surgery, Department of Surgery, Nationwide Children’s Hospital, The Ohio State University College of Medicine, Columbus, OH, USA)

Maria Marcela Bailez (Department of Pediatric Surgery, Hospital Garrahan Buenos Aires, Argentina)

Lohfa B Chirdan (Pediatric Surgery Unit, Department of Surgery, Jos University Teaching Hospital, Jos, PMB 2076, Jos, Nigeria)

Joep Derikx (Emma Children’s Hospital, Amsterdam UMC, location University of Amsterdam, Paediatric Surgery, Amsterdam, The Netherlands)

Shigehisa Fumino (Department of Pediatric Surgery, Kyoto Prefectural University of Medicine, Kyoto, Japan)

Nigel Hall (University Surgery Unit, Faculty of Medicine, University of Southampton, Southampton, UK)

Afua Hesse (Paediatric Surgery and Anatomy, Accra College of Medicine, Accra, Ghana)

Lieke Josephine van Heurn (Emma Children’s Hospital, Amsterdam UMC, location University of Amsterdam, Paediatric Surgery, Amsterdam, The Netherlands)

Lodewijk Willem Ernest van Heurn (Emma Children’s Hospital, Amsterdam UMC, location University of Amsterdam, Paediatric Surgery, Amsterdam, The Netherlands)

Shawn StPeter (Chair, Department of Surgery – Children’s Mercy Kansas City, Kansas City, USA)

Tutku Soyer (Department of Pediatric Surgery, Hacettepe University, Faculty of Medicine, Ankara, Turkey)

Jos Twisk (Department of Epidemiology and Data Science , Amsterdam Public Health Research Institute, Amsterdam UMC, Vrije Universiteit Amsterdam, De Boelelaan 1089a, 1081 HV, Amsterdam, The Netherlands)

Tianyou Yang (Department of pediatric surgical oncology, Guangzhou Women and Children's Medical Center, Guangzhou Medical University, Guangdong, China)

**Statistical Analysis**

Lieke Josephine van Heurn (Emma Children’s Hospital, Amsterdam UMC, University of Amsterdam & Vrije Universiteit Amsterdam, Department of P(a)ediatric Surgery, Amsterdam, The Netherlands), Jos Twisk (Department of Epidemiology and Data Science , Amsterdam Public Health Research Institute, Amsterdam UMC, Vrije Universiteit Amsterdam, De Boelelaan 1089a, 1081 HV, Amsterdam, The Netherlands)

**Local Investigators**

*Argentina;* Maria Marcela Bailez (Department of Pediatric Surgery, Hospital Garrahan Buenos Aires, Argentina), Siffredi Juan Ignacio (Department of Pediatric Surgery, Hospital Garrahan Buenos Aires, Argentina*),* Pablo Lobos (Hospital Italiano de Buenos Aires, Argentina)

*Austria;* Holger Till (Head of the Dept. of Paediatric and Adolescent Surgery, Medical University of Graz, Austria)

*Bangladesh;* Ashrarur Rahman Mitul (Bangladesh Shishu Hospital & Institute, Dhaka, Bangladesh)

*Belarus;* Olga Govorukhina (Center of Pediatric Surgery of Belarus), Natalya Prokopenya (Center of Pediatric Surgery of Belarus)

*Belgium;* Antoine De Backer (Universitair Ziekenhuis Brussel and Saffier Network for Rare Diseases in Pediatric Surgery, Belgium), Helena Reusens (Department of Pediatric Surgery, Hôpital des Enfants Reine Fabiola, Université Libre de Bruxelles),

*Brazil;* Simone de Campos Vieira Abib (Pediatric Oncology Institutute - GRAACC - Federal University of São Paulo, Brazil)

*Bulgaria;* Penka Peneva Stefanova-Peeva (Department of Pediatric surgery, University Hospital "St. George" and Medical University, Plovdiv, Bulgaria), Nadezhda Tolekova (Pediatric Surgery department of UMHATEM "N. I. Pirogov" Sofia, Bulgaria)

*Cameroon;* Mouafo Tambo Faustin (Yaounde Gynaeco-Obstetric and Pediatric Hospital-faculty of medecine and biomedical sciences- University of Yaounde Cameroon)

*Canada;* Jean-Martin Laberge (McGill University, Montreal Children’s Hospital – Shriners Hospital for Children Canada), Augusto Zani (Division of General and Thoracic Surgery, The Hospital for Sick Children, Toronto, ON, Canada), Richard J.B. Walker (Division of General and Thoracic Surgery, The Hospital for Sick Children, Toronto, Canada; Division of General Surgery, Department of Surgery, University of Toronto, Toronto, Canada)

*Chile;* Maricarmen Olivos Pérez (Hospital de Niños Dr. Roberto del Rio. Santiago, Chile), Marco Andrés Valenzuela (Hospital de Niños Dr. Roberto del Río. Santiago, Chile)

*China;* Yuanchao Shen (Department of Pediatric Surgery, Guangzhou Women and Children’s Medical Center, Guangzhou Medical University,, China), Tianyou Yang (Department of pediatric surgical oncology, Guangzhou Women and Children's Medical Center, Guangzhou Medical University, Guangdong, China), Yan Zou (Department of Pediatric Surgery, Guangzhou Women and Children's Medical Center, Guangzhou Medical University, Guangzhou,510623, China)

*Croatia;* Stanko Ćavar (Division pediatric surgery, University Hospital centre Zagreb, Croatia), Zenon Pogorelić (Department of Pediatric Surgery, University Hospital of Split and Department of Surgery, University of Split, School of Medicine, Croatia)

*Czech Republic;* Lucie Pos (Department of Paediatric Surgery, 2nd Faculty of Medicine, Charles University and University Hospital Motol, V Uvalu 84, Prague 5, 150 06, Prague, Czech Republic), Richard Skaba (Department of Paediatric surgery, 2nd Faculty of Medicine, Prague, Czech Republic)

*Denmark;* Peter Hjorth Jørgensen (Department of Pediatric Surgery, Rigshospitalet Copenhagen University Hospital, Copenhagen, Denmark)

*Egypt;* Amr Abdelhamid AbouZeid (Faculty of medicine, Department of Pediatric Surgery; Ain Shams University, Cairo, Egypt), Mahmoud Elfiky (Kasr Al Ainy Faculty of Medicine, Cairo University), Heba Taher (Pediatric Surgery Cairo University, Egypt)

*Estonia;* Matis Märtson (Tallinn Children's Hospital, Head of the Surgical Clinic, Tallinn, Estonia)

*Ethiopia;* Miliard Derbew (Medical Education Partnership Initiative Junior Faculty Project, School of Medicine, College of Health Sciences, Addis Ababa University, Ethiopia), Workye Molla Tigabie (St.Peter Specialized Hospital, Addis Ababa, Ethiopia)

*Finland;* Antti Koivusalo (New Children´s Hospital, (Section of Pediatric Surgery, University of Helsinki), Helsinki, Finland), Mikko Pakarinen (Section of Pediatric Surgery, Pediatric Liver and Gut Research Group, New Children’s Hospital, University of Helsinki and Helsinki University Hospital, Helsinki, Finland, and Department of Women’s and Children’s Health, Karolinska Institute, Stockholm, Sweden)

*France;* Olivier Abbo (Surgical Department, Hôpital des Enfants de Toulouse, France), Alexis Pierre Emmanuel Arnaud (Department of pediatric surgery, CHU Rennes, Univ Rennes, Rennes, France), Quentin Ballouhey (Department of Pediatric Surgery, Limoges University Hospital Center, Limoges, France) Francois Bastard (University Hospital, Angers, France), Anne Dariel (Pediatric surgery department, Hospital for sick children La Timone, Assistance publique des hôpitaux de Marseille, Marseille, France), Sabine Irtan (Sorbonne Université, Armand Trousseau Hopsital –Assistance Publique Hôpitaux de Paris, Paris, France), Jean Francois Lecompte (Pediatric Surgery Department, Hopitaux Pédiatriques de Nice CHU-Lenval, Nice, France), Guillaume Levard (Pediatric Surgery Department, University Hospital, Poitiers), Sabine Sarnacki (Department of Pediatric Surgery, Hôpital Necker-Enfants Malades - APHP GH Centre and Université de Paris Cité, Paris, France), Rony Sfeir (Pediatric Surgery Unit, University Hospital of Lille Jeanne de Flandre, Lille, France), Nicolas Vinit (Department of Pediatric Surgery and Urology, Necker-Enfants Malades Hospital, APHPand Université Paris Cité, Paris, France)

*Germany;* Anne-Sophie Holler (Department of Pediatric Surgery, Dr. von Hauner Children's Hospital, University Hospital, LMU Munich), Marietta Jank (Department of Pediatric Surgery, University Medical Center Mannheim, Heidelberg University, Mannheim, Germany), Martin Lacher (Head of Department of Pediatric Surgery, University of Leipzig, Leipizig, Germany), Oliver J. Muensterer (Department of Pediatric Surgery, Dr. von Hauner Children's Hospital, University Hospital, LMU Munich, Germany), Karin Rothe (Department of Pediatric Surgery, Charité Universitätsmedizin Berlin, corporate member of Freie Universität Berlin and Humboldt Universität zu Berlin, Germany), Udo Rolle (University Hospital Frankfurt/M., Department of Paediatric Surgery and Paediatric Urology)

*Ghana;* Afua Hesse (Paediatric Surgery and Anatomy, Accra College of Medicine, Accra, Ghana)

*Greece;* Zoi Lamprinou (P. & A. Kyriakou Children’s Hospital, Athens, Greece)

*Hungary;* Zsuzsanna Jakab (Hungarian Childhood Cancer Registry, 2nd Department of Pediatrics, Semmelweis University, Budapest, Hungary), Peter Vajda (Division of Paediatric Surgery, Medical School, University of Pécs, Hungary), Agnes Vojcek (Division of Pediatric Hematology and Oncology, Department of Pediatrics, University of Pécs Medical School, Pécs, Hungary)

*Hong Kong;* Kenneth KY Wong (Division of Paediatric Surgery, Department of Surgery, Li Ka Shing Faculty of Medicine, University of Hong Kong, Queen Mary Hospital, 102 Pokfulam Road, Hong Kong, Hong Kong)

*India;* Mohan K. Abraham (Department of Pediatric surgery and Pediatric urology, Amrita Institute Of medical sciences, Kochi, Kerala, India), Kirtikumar J Rathod (Department of Pediatric Surgery, All India Institute of Medical Sciences, Jodhpur, India), Shilpa Sharma (Department of Pediatric Surgery, All India Institute of Medical Sciences, New Delhi)

*Indonesia;* Gunadi (Pediatric Surgery Division, Department of Surgery, Faculty of Medicine, Public Health and Nursing, Universitas Gadjah Mada, Dr. Sardjito Hospital, Yogyakarta, Indonesia)

*Iran;* Mehdi Sarafi (Pediatric Surgery Research Center, Research Institute for Children’s Health, Shahid Beheshti University of Medical Sciences, Teheran, Iran), Ahmad Khaleghnejad Tabari (Pediatric Surgery Research Center, Research Institute for Children's Health, Shahid Beheshti University of Medical Sciences, Tehran, Iran)

*Iraq;* Muataz Al Ani (Ninevah college of Medicine, Alkhansaa teaching hospital, Mosul pediatric surgery centre, Iraq)

*Ireland;* Gavin Kane (Department of Paediatric Surgery, Children's Health Ireland at Crumlin, Dublin, Ireland)

*Israel;* Igor Sukhotnik (Department Pediatric Surgery, Tel Aviv Sourasky Medical Center, Tel Aviv University, Israel)

*Italy;* Stefano Avanzini (Pediatric Surgery Department, IRCCS Istituto Giannina Gaslini, Genoa, Italy), Pietro Bagolan (Area of Fetal, Neonatal, and Cardiological Sciences, Children's Hospital Bambino Gesù-Research Institute, Rome, Italy; Department of Systems Medicine, University of Rome "Tor Vergata", Rome, Italy), Piergiorgio Gama (Pediatric Surgery, Department of Women's and Children's Health, University of Padua, Padua, Italy), Riccardo Guanà (Pediatric and Neonatal Surgery Unit, Regina Margherita Children’s Hospital, Turin, Italy), Alessandro Inserra (Tor Vergata University of Rome, Director of the graduate school in Pediatric Surgery; Academic Director of Pediatric Surgery, Head of U.O.C.General and Thoracic Surgery, Bambino Gesu' Pediatric Hospital), Mario Lima (Pediatric Surgery Sant'Orsola Hospital, IRCSS, University of Bologna, Bologna, Italy), Antonino Morabito (Meyer Children's Hospital IRCCS Florence, University of Florence, Italy), Alessandro Raffaele (Department of Pediatric Surgery, Fondazione IRCCS Policlinico San Matteo, Pavia, Lombardia, Italy), Giovanna Riccipetitoni (Fondazione IRCCS Policlinico San Matteo, Pavia, Italy), Calogero Virgone (Pediatric Surgery, Department of Women's and Children's Health, University of Padua, Padua, Italy)

*Ivory Coast;* Yapi Landry Ake (Cocody Teaching hospital at Abidjan in Ivory Coast ; Department of Mother and Child health, pediatric surgery)

*Japan;* Shigehisa Fumino (Department of Pediatric Surgery, Kyoto Prefectural University of Medicine, Kyoto, Japan), Yoshiaki Hirohata (Department of Pediatric Surgery, Kyoto Prefectural University of Medicine, Kyoto, Japan), Maho Inoue (Department of Surgery, Saitama Children's Medical Center, Saitama, Japan), Yutaka Kanamori (Department of General Surgery, National Center for Child Health and Development, Tokyo, Japan), Naonori Kawakubo (Department of Pediatric Surgery, Graduate School of Medical Sciences, Kyushu University, Fukuoka, Japan), Takashi Sasaki (Department of Pediatric Surgery, Osaka City General Hospital, Osaka, Japan), Tomoaki Taguchi (Department of Pediatric Surgery, Graduate School of Medical Sciences, Kyushu University, Fukuoka, Japan), Tatsuro Tajiri (Department of Pediatric Surgery, Kyoto Prefectural University of Medicine, Kyoto, Japan), Hirofumi Tomita (Department of Surgery, Tokyo Metropolitan Children's Medical Center, Tokyo, Japan), Noriaki Usui (Department of Pediatric Surgery, Osaka Women's and Children's Hospital, Izumi, Japan)

*Latvia;* Mohit Kakar (Department of Pediatric Surgery, Riga Stradins University & Children's Clinical University Hospital, Riga, Latvia)

*Lithuania;* Vidmantas Barauskas (Vaikų chirurgijos klinika, Lietuvos sveikatos mokslų universiteto ligoninė Kauno klinikos, Eivenių g. 2 LT-50161, Kaunas)

*Macedonia;* Toni Risteski (University Clinic for Pediatric Surgery, Faculty of Medicine, Ss. Cyril and Methodius, University in Skopje, N Macedonia)

*Malaysia;* Dayang Anita Abdul Aziz (Paediatric Surgery Unit, Department of Surgery, Faculty of Medicine, Universiti Kebangsaan Malaysia, Kuala Lumpur, Malaysia), Mohd Yusran Othman (Hospital Tunku Azizah, Kuala Lumpur Women's and Children's Hospital, Malaysia)

*Mexico;* Jose Martin Palacios Acosta (Servicio de Cirugía Oncológica, Instituto Nacional de Pediatría, Mexico City, Mexico)

*Montenegro;* Marija Kolinovic (Pediatric Surgeon, Institute for children's diseases, Clinical Centre of Montenegro, Podgorica, Montenegro)

*Morocco;* Mohammed Oulad Saiad (Department of general pediatric surgery, Mother and child unit, university hospital Mohamed VI, Cadi Ayyad University, Marrakesh, Morocco)

*The Netherlands;* Robertine van Baren (Department of Surgery and Pediatric Surgery, University Medical Center Groningen, Groningen, Netherlands), Ivo de Blaauw (Department of Pediatric Surgery, Radboud University Medical Center, Amalia Children's Hospital, Nijmegen, The Netherlands), Joep P.M. Derikx (Emma Children’s Hospital, Amsterdam UMC, location University of Amsterdam, Paediatric Surgery, Amsterdam, The Netherlands), Wim G. van Gemert (Department of Pediatric Surgery, University Medical Centre Maastricht, Maastricht, the Netherlands), Ramon Gorter (Emma Children’s Hospital, Amsterdam UMC, location University of Amsterdam, Paediatric Surgery, Amsterdam, The Netherlands) Lieke J. van Heurn (Emma Children’s Hospital, Amsterdam UMC, location University of Amsterdam, Paediatric Surgery, Amsterdam, The Netherlands), L.W.Ernest van Heurn (Emma Children’s Hospital, Amsterdam UMC, location University of Amsterdam, Paediatric Surgery, Amsterdam, The Netherlands), Marijke E.B. Kremer (Department of Pediatric Surgery, University Medical Centre Maastricht, Maastricht, the Netherlands), Cornelius E.J. Sloots (Department of Pediatric Surgery, Erasmus MC Sophia Children’s Hospital, Rotterdam, The Netherlands), Jos Twisk (Department of Epidemiology and Data Science , Amsterdam Public Health Research Institute, Amsterdam UMC, Vrije Universiteit Amsterdam, De Boelelaan 1089a, 1081 HV, Amsterdam, The Netherlands), Marc Wijnen (Department of Pediatric Surgery, Radboud University Medical Center, Amalia Children's Hospital, Nijmegen, The Netherlands & Prinses Maxima MC), Rene M.H. Wijnen (Department of Pediatric Surgery and Intensive Care, Erasmus MC-Sophia Children's Hospital, Rotterdam, Netherlands), Marieke J. Witvliet (Department of Pediatric Surgery, University of Utrecht, Wilhelmina Children's Hospital, UMC Utrecht, Utrecht, The Netherlands)

*Nigeria;* Adesoji Ademuyiwa (Paediatric Surgery Unit, Department of Surgery, Faculty of Clinical Sciences, College of Medicine, University of Lagos. Idi Araba, Lagos, Nigeria; Paediatric Surgery Unit, Department of Surgery, Lagos University Teaching Hospital. Idi Araba, Lagos, Nigeria), Chris Bode (Paediatric Surgery Unit, Department of Surgery, Faculty of Clinical Sciences, College of Medicine, University of Lagos. Idi Araba, Lagos, Nigeria; Paediatric Surgery Unit, Department of Surgery, Lagos University Teaching Hospital. Idi Araba, Lagos, Nigeria), Lohfa B Chirdan (Pediatric Surgery Unit, Department of Surgery, Jos University Teaching Hospital, Jos, PMB 2076, Jos, Nigeria), Okechukwu Hyginus Ekwunife (Nnamdi Azikiwe University Teaching Hospital Nnewi, Nigeria), Justina Seyi-Olajide (Paediatric Surgery Unit, Department of Surgery, Lagos University Teaching Hospital. Idi Araba, Lagos, Nigeria), Aminu Muhammed Umar (Abubakar Tafawa Balewa University Teaching Hospital, Bauchi, Nigeria)

*Norway;* Kristin Bjornland (Department of pediatric surgery, Oslo University Hospital, postboks 4950 Oslo, Norway and University of Oslo)

*Pakistan;* Muhammad Arshad (Professor of Pediatric surgery, Liaquat National Hospital and Aga khan university, Karachi Pakistan), Mohammad Ajad Chaudhry (Department of pediatric surgery, Children Hospital, Shaheed Zulfiqar Ali Bhutto Medical University, Islamabad, Pakistan), Muhammad Bilal Mirza (University of Child Health Sciences Lahore Pakistan)

*Philippines;* Beda Espineda (Philippine Children's Medical Center as a Senior Consultant in Department of Pediatric Surgery), Maria Celine A. Villegas (Chief, Division of Pediatric Surgery, Department of Surgery, University of the Philippines - Philippine General Hospital; Associate Professor, University of the Philippines College of Medicine, University of the Philippines Manila)

*Poland;* Weronika Jaron (Department of Pathology, The Children's Memorial Health Institute, Warsaw, Poland), Piotr Kalicinski (Department of Pediatric Surgery and Organ Transplantation, Children's Memorial Health Institute, 04-730 Warsaw, Poland), Maciej Murawski (Department of Pediatric Surgery and Urology, Medical University of Gdansk, Poland)

*Portugal;* Rui Alves (Pediatric Surgery Department, Hospital Dona Estefania, Lisboa, Portugal), Maria Carolina Sobral (Hospital Dona Estefânia - Centro Hospitalar Universitário Lisboa Central)

*Romania;* Vlad-Laurentu David (Department of Pediatric Surgery and Orthopedics, "Victor Babes" University of Medicine and Pharmacy Timisoara, Romania)

*Russia;* Alexey A. Gusev (FSAI "NMRC of Сhildren Health" MHRF, Moscow, Russian Federation; RUDN University, Moscow, Russian Federation), Khvorostov I.N. (Volgograd State Medical University), Yury Kozlov (Irkutsk Regional Children’s Hospital, Director of Regional Center for Pediatric Minimally Invasive Surgery and Pediatric Robotics, Irkutsk, Russia; Department of Pediatric Surgery, Irkutsk State Medical Academy of Postgraduate Education, Russia; Department of Pediatric Surgery and Pediatrics, Irkutsk State Medical University, Russia), Minaev Sergey Viktorovich (Department of pediatric surgery of Stavropol State Medical University, Russia)

*Serbia;* Maja Milickovic (Department of Abdominal Surgery, Institute for Mother and Child Healthcare of Serbia "Dr Vukan Cupic", Belgrade, Serbia), Sanja Sindjic-Antunovic (University Children’s Hospital, Center for Pediatric Surgery and Medical Faculty University of Belgrade, Serbia)

*Singapore;* York Tien Lee (Senior Consultant, Department of Paediatric Surgery, KK Women’s and Children’s Hospital, Singapore; Clinical Assistant Professor, DUKE-NUS Medical School, Singapore)

*Slovakia;* Rebeka Pechanová (National Institute of Children´s Diseases, Bratislava, Slovakia)

*Slovenia;* Jože Maučec (Department for Pediatric Surgery, University Medical centre Ljubljana, Slovenia)

*South Korea;* Hyun-Young Kim (Department of Pediatric Surgery, Seoul National University Hospital, 101 Daehak-ro, Jongno-gu, Seoul, 03080, Republic of Korea; Department of Pediatric Surgery, Seoul National University College of Medicine, Seoul, Republic of Korea), Seong Chul Kim (Department of Pediatric Surgery, University of Ulsan College of Medicine and Asan Medical Center, Seoul, Korea), Sohyun Nam (Division of Pediatric surgery, Department of Surgery, Inje University Busan Paik hospital, Busan, South Korea)

*Spain;* Gabriela Guillén (Department of Pediatric Surgery, University Hospital Vall d´Hebron, Barcelona, Spain, Leopoldo Martinez (General-Oncologic Pediatric Surgery, Children's Hospital La Paz, Spain)*,* Maria Molina (Virgen del Rocio Children's Hospital, Department of Pediatric Surgery, Sevilla, Spai), Fernando Vázquez Rueda ( Pediatric Oncologic Surgery, Pediatric Surgery Service, Hospital Universitario Reina Sofía. Córdoba, Spain), Oscar Girón Vallejo (Pediatric Surgery Department. Pediatric Surgical Oncology, Virgen de la Arrixaca University Clinical Hospital, Murcia, Spain), Maria Bordallo Vazquez (Pediatric Surgery Department. Pediatric Surgical Oncology Unit. La Fe University and Polytechnic Hospital, Valencia, Spain)

*Sri Lanka;* Naveen Wijekoon (Department of Surgery, University of Colombo, Consultant Paediatric Surgeon - Lady Ridgeway Hospital, Sri Lanka)

*Sweden;* Mette Hambraeus (Department of Pediatric surgery, Skane University Hospital Lund, Lund, Sweden), Helene Engstrand Lilja (Department of Women’s and Children’s Health | Karolinska Institutet, Unit of Pediatric Surgery | Karolinska University Hospital), Par-Johan Svensson (Consultant Paediatric Surgeon Department of Paediatric Surgery, Astrid Lindgren´s Childrens Hospital, Karolinska Hospital and Karolinska Institute, Stockholm, Sweden), Tomas Wester (Karolinska University Hospital, Stockholm, Sweden)

*Syria;* Husam Dalati (Department of Pediatric Surgery, Children Hospital, Damascus, Syria), Houssain Al Halabi (Department of Pediatric Surgery, Children Hospital, Damascus, Syria), Qusai Mashlah (Department of Pediatric Surgery, Children Hospital, Damascus, Syria)

*Taiwan;* Shih-Hsiang Chen (Division of Hematology/Oncology, Department of Pediatrics, Chang Gung Memorial Hospital, Chang Gung University College of Medicine, Taoyuan, Taiwan)

*Thailand;* Montinee Supchatura (Department of Surgery, Queen Sirikit National Institute of Child Health, Bangkok, Thailand)

*Tunisia;* Saloua Ammar (Département of Pediatric surgery, Hedi Chaker Hospital University of medecine of Sfax, University of Sfax, Tunisia), Salma Mani (Département of Pediatric surgery, Fattouma Bourguiba Hospital, University of medecine of Monastir, Tunisia)

*Turkey;* Orkan Ergün (Ege University Department of Pediatric Surgery, Izmir, Türkiye), Cigdem Ulukaya Durakbasa (Department of Pediatric Surgery, Istanbul Medeniyet University Faculty of Medicine), Samir Hasan (Ege University Department of Pediatric Surgery, Izmir, Türkiye), Ayşe Karaman (University of Health Sciences Turkey, Dr Sami Ulus Maternity and Children Health and Research Application Center, Department of Pediatric Surgery), İbrahim Karaman (University of Health Sciences Turkey, Dr Sami Ulus Maternity and Children Health and Research Application Center, Department of Pediatric Surgery), Tezer Kutluk (Department of Pediatric Oncology, Hacettepe University, Faculty of Medicine & Cancer Institute Ankara, Turkey), Tutku Soyer (Department of Pediatric Surgery, Hacettepe University, Faculty of Medicine, Ankara, Turkey)

*Ukraine;* Yevhen Rudenko (Department of Pediatric Surgery, Bogomolets National Medical University, Neonatal Surgery unit, National Specialized Children Hospital “Ohmatdyt“, Kyiv, Ukraine)

*United Kingdom;* Sarah Braungart (Department of Paediatric Surgery, Leeds Teaching Hospitals, UK), Deepika Bhojwani (Department of paediatric surgery, Cambridge University Hospitals NHS Foundation Trust), Alison Morag Campbell (Department of paediatric surgery, Great North Children's Hospital, Newcastle), Manal Dhaiban(FRCS England , MS Paediatric surgery, Speciality Registrar Paediatric surgery in Birmingham Children hospital UK), Paul Farrelly (Department of Paediatric Surgery, Royal Manchester Children's Hospital, Manchester, UK), Florian Friedmacher (Department of Pediatric Surgery, The Royal London Hospital, London, United Kingdom; Department of Pediatric Surgery, University Hospital Frankfurt, Goethe University Frankfurt, Germany), Robin Garrett-Cox (Consultant Paediatric Surgeon at Bristol Royal Children's Hospital, Bristol UK), Stefano Guiliani (Developmental Biology and Cancer Programme, UCL Great Ormond Street Institute of Child Health, London, UK; Department of Specialist Neonatal and Paediatric Surgery, NHS Foundation Trust, Great Ormond Street Hospital for Children, London, UK), Nigel Hall (S University Surgery Unit, Faculty of Medicine, University of Southampton, Southampton, UK), Paul Losty (Professor of Paediatric Surgery, Institute Of Life Course And Medical Sciences, University Of Liverpool, UK), Jonathan Neville (Department of Paediatric Surgery, University Surgery Unit, University Hospitals Southampton), Kathryn O'Shea (Royal Manchester Children's Hospital, Manchester, UK), Rebecca A Roberts (Department of Paediatric Surgery and Urology, Bristol Royal Hospital for Children, Bristol), Mohamed Shalaby (Department of Pediatric Surgery, Royal Hospital for Sick Children, Edinburgh, United Kingdom), G. Suren Arul (Consultant paediatric surgeon, Birmingham Children’s Hospital, Birmingham, UK)

*United States;* Jennifer H. Aldrink (Division of Pediatric Surgery, Department of Surgery, Nationwide Children’s Hospital, The Ohio State University College of Medicine, Columbus, OH, USA), Zachary Kastenberg (Division of Pediatric Surgery, Department of Surgery, University of Utah School of Medicine, Salt Lake City, USA), Maria E. Knaus (Department of Surgery, University of Tennessee Health Science Center), Bethany J. Slater (Department of Surgery, University of Chicago, Chicago, IL), Shawn StPeter (Chair, Department of Surgery – Children’s Mercy Kansas City, Kansas City, USA)

*Zambia;* Bruce Bvulani (Department of Paediatric Surgery, University Teaching Hospital of Lusaka, Lusaka, Zambia)
